# Pediatric Surgery and Anesthesia in Low-Middle Income Countries: Current Situation and Ethical Challenges

**DOI:** 10.3389/fped.2022.908699

**Published:** 2022-07-28

**Authors:** Rebecca Pulvirenti, Marianna Gortan, Dioniso Cumba, Piergiorgio Gamba, Costanza Tognon

**Affiliations:** ^1^Pediatric Surgery Unit, Department of Woman's and Child's Health, Padua University Hospital, Padua, Italy; ^2^Department of Surgery, Pediatric Hospital of Sao José Em Bor, Bissau, Guinea-Bissau; ^3^Anesthesiology Pediatric Unit, Department of Woman's and Child's Health, Padua University Hospital, Padua, Italy

**Keywords:** low-middle income countries (LMICs), pediatric surgery, Global Surgery, humanitarian mission, ethics, global health

## Abstract

Low-middle income countries (LMICs) are currently experiencing an important population growth, leading to a substantial raise in the number of children living in those areas. As a consequence, the existing gap between the need for surgical and anesthetic care and the available therapeutic options will increase. To overcome this, an improvement in the available expertise, infrastructures, and supplies will be mandatory. The implementation of educational and training programs for local healthcare providers should be a top priority. Alongside, the population's awareness on the necessity to seek for medical care should be deployed, together with an eased access to health facilities. Based on the existing literature and our 20-years' experience in humanitarian missions, our article aims to investigate the status of pediatric surgery in LMICs, and the role of western aids in the implementation of this ever-increasing field of expertise.

## Introduction

Over the past decades, several humanitarian missions have been deployed toward low-middle income countries (LMICs), playing a key role for healthcare in non-conflict, war or natural disaster settings.

The access to healthcare facilities is taken for granted in most high-income countries (HICs), where the healthcare systems are set up with adequate infrastructures and facilities. When it comes to humanitarian missions for LMICs, a whole different scenario needs to be considered. The paucity of instruments, drugs, and trained staff are common issues, which can be overcome with proper missions' planning. Independently from the mission's purpose, ethical dilemmas are always on the rise, and represent one of the main challenges. Indeed, the real limit of such initiatives is that they are built to address medical care to a restricted number of people, and require specialists to choose who is feasible to help over as many needy patients. Adding up to the lack of funding and resources, this and other ethical considerations are particularly challenging in the fields of obstetrics and pediatrics.

Our experience with humanitarian missions consists in a 20-years' relationship with Sao José Em Bor Hospital, a pediatric tertiary referral center in Guinea Bissau. Surgical missions comprehended a 2-weeks period of in-place work once or twice a year, preceded by an accurate surgical planning together with the local staff. Unfortunately, over the last 2 years, the deployment of humanitarian aids was not possible due to the SARS-CoV-2 pandemic. Nevertheless, we keep on providing remote assistance and training, aiming to restart the in-place work as soon as possible.

In this article we aim to analyze the reasons which turn the provision of healthcare in LMICs an open challenge and the ethical dilemmas behind humanitarian aids, providing inputs from our front-line experience.

## Pediatric Surgery in LMICs

For many years, surgery has been neglected in global health programs addressed to LMICs (1). Just recently, important initiatives, such as the Lancet Commission on Global Surgery (2015), the introduction of a specific volume “Essential Surgery” in the third edition of “Disease Control Priorities in Developing Countries” (DCP) (2015) and the World Health Assembly (WHA) Resolution on Emergency and Essential Surgical Care (2015); pointed out the lack of surgical services in LMICs and the necessity to scale them up (2). Based on the esteems of the Lancet Commission on Global Surgery, five billion people worldwide do not have access to surgical and anesthetic care, when needed. In particular, surgical procedures are precluded to 93% of the population in Sub-Saharan Africa and to 97% of the population in Southern Asia (1). Based on the data of DCP-3, 1.5 million deaths per year could be avoided with the provision of adequate and safe surgical procedures (3).

The burden of pediatric surgical conditions in LMICs still remains undetermined (4). Children and adolescents represent 47% of the African population, which is constantly and actively expanding. In fact, while nowadays 580 millions children live in Africa, representing 25% of the global pediatric population, it is expected that almost one billion children will live in Africa by 2050, accounting for 40% of the world pediatric population (5, 6). Despite this trend, little attention has been given to surgical conditions affecting children in LMICs. The few published studies on the subject pointed out that pediatric surgical conditions are frequent, and often do not receive adequate treatment; this translates in higher rates of premature deaths and disability (7). By the end of last century, Bickler et al. estimated that the cumulative risk of needing a surgical procedure before 15 years of age in Banjul (Gambia) was 85.4% (8). More recently, Butler et al. tried to estimate the necessity of pediatric surgical procedures in LMICs through a randomized survey undertaken in Rwanda, Sierra Leone, Nepal and Uganda. They found that 19% of children presented a disease requiring surgical treatment. Moreover, 62% of them had at least one unmet surgical need, namely a condition which could be treated with a surgical procedure and for which the patient did not seek for medical assistance or sought for it but did not receive it (4). Lastly, Gupta et al. found that 7% of all unmet surgical needs in Nepal was due to congenital anomalies (9). Accordingly, analyzing the causes of worldwide under-five mortality (mortality that is 15-folds superior in Sub-Saharan Africa than in Europe - 78 children every 1,000 live births), it is evident that two groups of surgical conditions - congenital anomalies and traumas - are responsible for 9 and 7% of deaths, respectively (10).

## Causes Behind the Difficult Access to Surgical Care in LMICs

Access to surgical care in LMICs is limited by several factors, including shortage of workforce and facilities, local traditions, and poor resources. Several humanitarian aids are being deployed to face some of these difficulties, notwithstanding the need to increase the local authorities involvement.

### Poor Investments

Historically, little investments have been made to guarantee surgical assistance in LMICs. For a long time, surgery has been considered a luxury in these areas - a too expensive and non-essential service (11). Available resources have been preferably used in vaccination campaigns, in the fight against HIV and in other public health interventions (12). Without diminishing the value of such interventions, it should be considered that in 2010, for example, about 16.9 million people died due to surgical conditions; a number that overcomes the deaths for HIV/AIDS (1.46 million), tuberculosis (1.20 million), and malaria (1.17 million) altogether (13, 14). Moreover, the assumption that surgery is a too expensive service in relation to its efficacy has been disproved by several studies. For what concerns pediatric surgery, a first cost-efficacy analysis was conducted by Gosselin et al. in Sierra Leone, using DALYs (Disability-Adjusted Life Years). From this analysis emerged a positive impact of surgery, comparable to that of other, far more financed public health interventions (15). The Lancet Commission on Global Surgery reaffirms this concept and states that investing in surgical services is economically accessible, saves lives and promotes economic growth. Without a scale-up of the investments in the surgical field, LMICs countries will continue to experience losses in the economic productivity; losses estimated to be around 12.3 trillion dollars between 2015 and 2030 (1).

Humanitarian missions always aimed to provide medical or surgical aids in low-income areas, with the idea of compensating the inherited paucity of infrastructures and workforce. For the duration of the mission, healthcare services can count on external provisions, and several treatments can be carried out because of exceptional material availability. As a consequence, many people can be helped by the visiting staff. Yet, once the mission is over, both, patients and clinicians, will face pre-mission conditions. This is a fundamental aspect to consider when planning humanitarian interventions.

**Figure 1:**
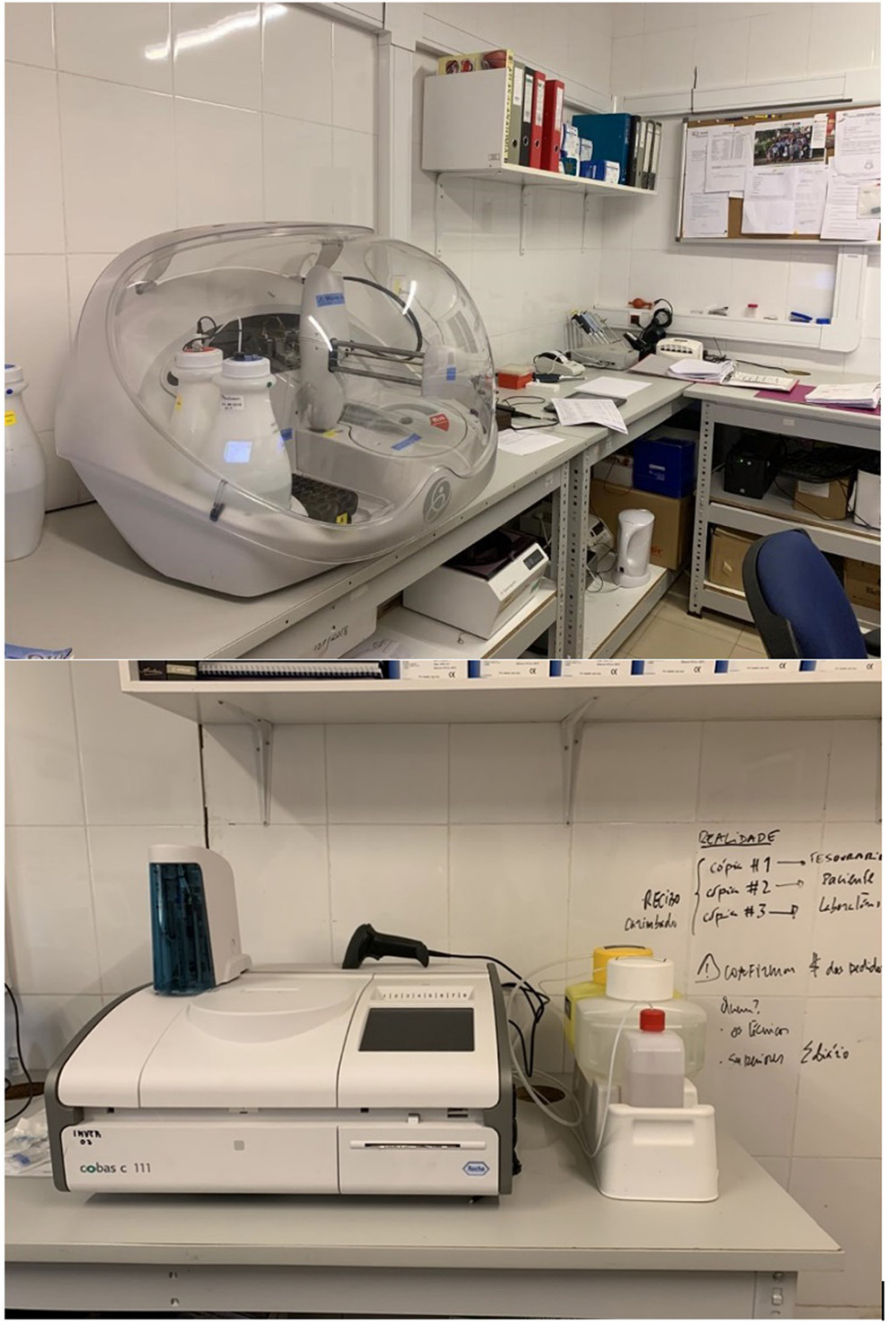
Initial setting of the laboratory for blood analysis and transfusions.

Despite the lack of supplies is not an easily solvable issue, the visiting group should aim to bring some long-lasting resources, and work together with local authorities to combine visiting centers' and local's investments. This is one of the key points we considered during our humanitarian missions in Guinea Bissau. Thanks to the commitment of the local staff and authorities, it was possible to work together in order to create a previously non-existent laboratory for blood analysis and transfusions. The structure is still running and resulted to be an essential implementation for the clinicians' daily work (Figure 1).

### Shortage of Medical Staff

There is a non-negligible paucity of surgical specialized staff in LMICs. Indeed, only 20% of the global healthcare workforce is covering areas where the 48% of the global population lives. More specifically, only 19% of surgeons, 15% of anesthesiologists and 29% of obstetricians work in LMICs (16). Just 12% of the global surgeons work in Africa and Southern Asia, where one third of the global population lives. This results in an estimated need for 1.27 million SAO (surgeons, anesthesiologists and obstetricians) providers by 2030 (1). A study conducted by the College of Surgeons of East, Central and Southern Africa (COSESCA) calculated a ratio of 0.53 surgeons per 100.000 inhabitants (with a minimum of 0.18 per 100.000 inhabitants in Burundi), in his region of interest - namely Burundi, Ethiopia, Kenya, Malawi, Mozambique, Ruanda, Tanzania, Uganda, Zambia and Zimbabwe -; even though without considering the specialists in Gynecology and Obstetrics, Ophthalmology, and those surgeons who operate in these countries on occasional basis (17). Despite the limitations of this study, the picture presented by COSESCA consistently differs from the minimum target theorized by the Lancet Commission on Global Surgery, recommending at least 20 surgeons per 100.000 inhabitants (1). As a comparison, in the U.S.A a ratio of 54.71 surgeons per 100.000 inhabitants is found and reaches 95.7 surgeons per 100.000 inhabitants in the Euro Area (18). Referring to pediatric surgery, a study conducted in 2017 and evaluating the current status of this specialty in 26 African nations, found a ratio of 0.26 pediatric surgeons per 1.000.000 inhabitants. This implies the need for 3.300 additional pediatric surgeons in these nations, to reach the minimum ratio of one pediatric surgeon per 100.000 children, as theorized by the American Pediatric Surgical Association (APSA) (19). Moreover, it should be considered that the majority of pediatric surgeons work in cities, leaving the rural areas without surgical coverage. More specifically, the COSESCA study found that 71% of surgeons work in cities with more than 500.000 inhabitants (17). The small number of pediatric healthcare providers operating in rural areas translates in the affirmation of the, so called, essential surgeons. Indeed, in LMICs setting, SAO providers are single doctors capable of handling different surgical conditions and procedures.

The reasons behind the paucity of specialized pediatric workforce in LMICs are several, yet the lack of standardized pediatric anesthesia and surgical training programs is one of the leading. Not only training programs are missing, but the ones available greatly differ even within the same country. Chirdan et al., based on their survey, reported that 22 out of 49 African centers with a pediatric surgery team have irregular or no trainees per year or session of training (20). Pediatric surgeons across LMICs need to be more involved in medical students or junior trainees mentoring to help increase the enrollment in pediatric surgical training. To fill the gap between the available pediatric surgical care and the population's needs, Government and institutional involvement is crucial. Considering these data, it is evident that the shortage of surgical staff cannot be solved - at least in the next few years - just through the training of new surgeons, but should also rely on providing more basic surgical competences to generalist surgeons or health workers, as well as more pediatric surgical competences to general surgeons (3, 11).

General training of local surgeons would not only fill the workforce gap, but would lead to a more cost-effective system; indeed, surgery performed by locals require lower costs and grants proper patients' follow-up. Additionally, it represents the basis for LMICs codevelopment.

Another well-known issue refers to the local workforce migration toward developed countries. Lantz et al. estimated than 12.8% and 12.1% of surgical specialists who graduated, respectively, in Africa and South East Asia work in HICs (21). This percentage is estimated to be even higher when adding up medical students to the number of newly-graduated doctors or consultants. Indeed, most LMICs count on a private education, where local trainees or medical students need to pay in order to be trained. As a result, wealthy people seldom choose to continue with their education abroad, looking for better reward or remuneration. Hagander et al. investigated the main drivers of LMICs brain drain; they circulated an internet-based nationwide survey among surgeons living in the United States (US) and who originated from LMICs, finding out that the most influential factors for primary migration were related to professional reasons. Non-professional factors, such as concern for remuneration, family, and security were significantly less important for the initial migration decisions, but adopted a more substantial role in deciding whether or not to return after training in the US (22). The establishment of structured and specific training paths and initiatives to implement surgical infrastructures might help avoid such trend, and, in the mid-long run, partially implement the available workforce.

Alongside the brain drain phenomenon, LMICs are facing a progressive workforce migration from rural areas to urban districts, aiming to leave behind the burden of cross-specialty surgical interventions. Poor work and family conditions are bringing an ever-increasing number of surgeons to move toward bigger hospitals, where they can focus on highly-specialized surgeries without working round-the-clock (23).

Referring to perioperative care, anesthesia is frequently administered by trained nurses, and no pediatric staff is available. Maman et al. reported the presence of one anesthesiologist and one anesthetic nurse per 800.000 and 100.000 inhabitants, respectively, in thirteen French-speaking sub-Saharan African countries (24). Again, most anesthesiologists are located in cities, while rural areas can count on a limited number of anesthesia nurses. According to their study, up to 50% of the anesthesia nurses work without medical supervision, and 20% of them only received on-site training. This translates in an extremely reduced manpower able to face complex conditions, such as major congenital anomalies or major surgeries, and many unnecessary perioperative deaths. A study from Talabi et al. reported mortality rates within 24 h and 30 days after surgery rounding up to 34 per 10.000 and 56 per 10.000 procedures, respectively (25). Such rates were negatively impacted by the decreasing age of the patients, with higher levels involving infants with congenital anomalies, who require adequate staffing and monitoring during the perioperative period. Skilled anesthesia providers and appropriate resources are required to avoid anesthesia-related perioperative deaths.

During our humanitarian missions in Guinea Bissau, a super-posable situation was found, with no specialized or highly-trained staff available. Except for the chief of department, doctors only had access to medical school and did not receive specific pediatric surgery training. No (pediatric) anesthesiologists were available and perioperative care was handled by nurses. This not only translated in non-optimal patient care, but also in the absence of perioperative planning or creation of surgical and anesthesia registries. During the stay in the hosting center, efforts were made both to perform complex surgeries, and to train the local staff on basic and standard procedures. According to resources and drugs' availability, major relevance was given to post-operative care. Nurses were taught on how to evaluate children's parameters after surgery and to control pain, in order to decrease the length of stay. Additionally, operative registries were introduced and their use for patients' follow-up was stressed. Nevertheless, perseverance in their usage is not always easy and this instrument is still not fully incorporated into daily activities.

### Costs

Individual costs are one of the main limiting factors to the access to surgical care. It is estimated that, every year, 33 million people experience a catastrophic expense to pay for surgical care. Moreover, additional 48 million people face a financial disaster because of the non-hospital expenses related to surgical treatment (transports, food, accommodation) (1).

In 1978 the famous Declaration of Alma Ata firstly affirmed that “*Governments have a responsibility for the health of their people (...). A main social target of governments, international organizations and the whole world community in the coming decades should be the attainment by all peoples of the world by the year 2000 of a level of health that will permit them to lead a socially and economically productive life. Primary health care (PHC) is the key to attaining this target”* “*PHC is essential health care (…) made universally accessible to individuals and families in the community (…) at a cost that the community and country can afford”* (26). Another concept which is strongly related to PHC is Universal Health Coverage (UHC), namely the deployment of essential health services to all individuals, with a special emphasis on the poor, vulnerable, and marginalized segments of the population; while ensuring that the use of these services does not expose the users to financial hardship. In order to achieve UHC, the UN general assembly on Global Health and Foreign Policy (2012) called upon “*Member States to ensure that health financing systems evolve so as to avoid significant direct payments at the point of delivery and include a method for prepayment of financial contributions for health care and services as well as a mechanism to pool risks among the population in order to avoid catastrophic health-care expenditure and impoverishment of individuals as a result of seeking the care needed”*^1^.

Despite these resolutions, many countries' health systems continue to be inequitable. Backman et al. (27) identified in their work some indicators that reflect the right-to-health features of health systems and collected data on these indicators for 194 countries. Accordingly to their data, only 63 countries recognize in their constitution or other statute the right to health.

As not even medical essential services are universally guaranteed, the lack of national health and UHC plans make surgery still economically inaccessible for a large portion of the global population. Okoroh et al. (28) conducted a systematic review to provide a comparative analysis of the inclusion of surgical care in operating plans for UHC in LMICs. They found out that, even if surgery was included in many UHC policies, 80% of the analyzed countries had an out-of-pocket expenditure of 60 % or more, making surgical services, even if partially covered, largely inaccessible.

As to the pediatric patients, not only hospital direct (the cost of surgical intervention) and indirect (food, transportation, accommodation) expenses should be considered, but also emphasis should be given upon the money drain related to the loss of working days of the caregiver. In this perspective, lots of families in LMICs would seek for surgical care only with advanced medical conditions, as the loss of working days (especially in some periods of the year, as the harvesting season), associated to the costs of healthcare and transportation, would cause an economic catastrophe.

With regards to our humanitarian experience, Sao José Em Bor Hospital examinations at the outpatient clinic are free-of-charge, while an economic contribution is asked for surgical interventions. Whenever the family cannot afford surgical treatment, an Italian non-profit organization would cover the surgical expenses.

Since 2015, the development of cost-effective plans aiming to implement the access to surgical, obstetric and anesthesia (SAO) services has become an area of interest both for healthcare professional associations and government institutions. Mutual commitment brought to the definition of National Surgical, Obstetric and Anesthesia Plans (NSOAPs), together with practical recommendations on how to implement such plans into each country health policy (29). Through an extensive evaluation of the burden of surgical diseases, tailored plans prioritizing country-specific needs are under development. Traumas, pediatric surgical conditions, and anesthesia care are main priorities of national plans. Alongside, raising the institution's awareness on the cost-effectiveness of surgical procedures and the mid- long-term positive effect of SAO services on the population's well-being is the starting point for healthcare access implementation. As initial result of this ongoing process, Nepal and Pakistan have now completed the NSOAP development process and have been working together with several stakeholders and national government to integrate surgical planning into the national health strategy (30, 31).

### Distances

As previously mentioned, surgical services mostly concentrate in large central hospitals, obliging people to walk long distances before receiving adequate surgical care. Long distances and costs of transportation prevent children from receiving timely treatment for their diseases. Moreover, many children are brought to medical or surgical attention only after visiting local traditional illers. As a consequence, patients often arrive at the hospital with a life-threatening advanced disease (32). Another consequence of this scarce access to surgical care is that medical conditions, which would not necessitate surgical intervention in areas with a structured first level sanitary system, can progress till becoming of surgical pertinence, as in the case of osteomyelitis (1).

To overcome the problem of distances and reach rural areas, a group from Ghana instituted a mobile clinic. A group of five professionals, namely a pediatric surgeon, nurse, ward assistant, pharmacist, and a trained pediatric surgical medical assistant; moved regularly with a jeep among ten health-spots to offer basic surgical care (33). As a result, surgical conditions could be treated at early stages, thus diminishing associated morbidities and complications.

When referring to emergency conditions, HICs can count on high-end ambulance systems. This scenario cannot be transported to LMICs, in which roads and means of transportation are frequently far from western standards. Context-specific appropriate solutions need to be implemented to increase the population's chances to reach adequate care. One example is the BRAC model from Bangladesh, through which patients can be brought to dedicated pick-up points for state ambulance transportations (23).

### New Perspectives

As more and more attention is given to the empowerment of LMICs, several organizations are now working in order to provide tools and pathways to implement essential surgical care into national healthcare systems. Both, the DCP-3 and NSOAP manual, aim to supply LMICs with technical assistance and capacity building for priority setting, development and implementation of health services (29, 34). In this regards, a key role is given to SAO activities, with special focus on congenital anomalies and perioperative anesthesia care. Alongside, the Global Initiative for Children's Surgery (GICS) is gathering expertise from LMICs and high-income countries (35); representatives of both counterparties developed several projects focusing on the implementation of infrastructures, service delivery, training and local research (36).

The first step into SAO services implementation require a combined effort between local facilities and surgical missions. From one side, the improvement of existing structures capacity with training of local staff and development of surgical protocols; while, from the other side, well-planned humanitarian missions addressing local needs and highly-prevalent conditions. In this context, a close partnership between academic organizations and development programs will be needed.

The provision of clinical intervention at every healthcare level is an additional step. As stated in the DCP-3, services provided by different infrastructures should be rearranged in order to ensure basic surgical assistance even at village health centers level (37). As a result, distances and late access to healthcare could be partially overcome.

Lastly, the ever-increasing spreading of advanced technology might help the provision of specialized workforce assistance. Distances and costs could be beaten by a real time communication between local professionals and third-level facilities experts, who may guide patient treatments and avoid the development of life-threatening conditions (37).

## Humanitarian Missions

Based on the Office for the Coordination of Humanitarian Affairs (OCHA), Humanitarian missions are defined as “Aid that seeks to save lives and alleviate suffering of a crisis-affected population.” In this view, an accurate planning of humanitarian missions is fundamental for them to be effective. Local institutions and surgeons should be committed to coordinate HICs' efforts and define potentially long-lasting interventions.

Since the very beginning, humanitarian missions presented a standard pattern, being time-limited, influenced by the local political, social and cultural situation; and hampered by linguistic barriers (38).

Recently, the American Academy of Pediatrics (AAP) Delivery of Surgical Care Subcommittee and the American Pediatric Surgical Association (APSA) Global Pediatric Surgery Committee, together with the American Pediatric Surgical Nurses Association, Inc. (APSNA) Global Health Special Interest Group and the Society for Pediatric Anesthesia (SPA) Committee on International Education and Service, generated consensus recommendations for short-term missions (39). Nevertheless, the uniqueness of every mission needs to be considered and general guidelines might not always be applicable to the local situation.

A well-planned intervention should not only provide surgical care for the affected children, but also raise the awareness of the potential impact of a structured facility on children's health. Anyhow, defining long-term outcomes of such missions is challenging, also due to local struggling and difficulties to identify measurable outcomes. Benchmarks of a fruitful humanitarian mission should be the development of consolidated infrastructures, the facilitation of local staff training and the implementation of access to pediatric surgical care, reducing the reliance on foreign help (40).

To ensure an effective mission, several factors need to be considered.

First of all, the different setting in which western doctors will operate needs to be taken into account. Surgical plans should be tailored on the necessities of the Country toward which they are directed. As pointed out by doctor Diana Farmer in her Presidential address given at the 48th Annual meeting of APSA (American Pediatric Surgical Association), lots of well-intentioned initiatives to empower children's surgical care in low-income countries fail as they are directed by experts who operate in high-income countries and do not know which are the actual needs of Low-income countries (32).

Different countries are working in order to adapt their aids to the local situation. Hubertus et al. reported their experience with the development of a pediatric surgery unit in Ethiopia, able to offer patient care within local conditions and independent from external aids (41). Training of future fellows was also a purpose. The visiting team provided pediatric training to two general surgeons, covering surgical and management skills. The whole mission resulted in a working pediatric surgery unit, providing specialized care in an otherwise unserved area.

An additional point to mention of this work refers to the retrieval of pediatric surgery tools. While in the initial phases the visiting team provided the new unit with facilities, it was soon clear that this approach was not fruitful. Indeed, free material supply prevents the development of a dedicated infrastructure for the purchase and maintenance of material. Moreover, local suppliers are not able to compete with a free of charge acquisition. Lastly, donated surgical equipment might be technologically advanced, according to HICs' supplies. This may turn into a scarce use of such material due to lack of expertise and, in case of damage, no possibilities to fix it. Supplies should always match the technology of the local environment. Moreover, as Roy stated, addressing the equipment maintenance, after adequate training, to locals could provide employment, sense of pride and well-functioning instruments (23).

Another good example of how surgical delivery should be adapted to the local conditions is offered by Olivieri et al. This group reported the use of a modified technique for the posterior sagittal anorectoplasty (PSARP), aiming to improve the postoperative course of patients with ano-rectal malformations (42). In their experience, scarce nursing resources and limited hospitalization were the main determinants for the ineffectiveness of the surgical procedure. Indeed, starting in 2009, a small change to the usual PSARP technique has been implemented, leaving a long rectal stump anchored to the perineal skin in order to protect the wound from the anal mucous discharge. This exemplifies how the visiting team's ability to integrate with the local conditions is crucial for a satisfactory result of a mission. It also relates to the uselessness of focusing humanitarian missions on treating complex and ultra-specialized conditions, as this may leave local staff in the position of handling complications of complex surgeries once the mission is over. This would not only be unhelpful, but would also increase the number of unnecessary perioperative deaths.

Providing adequate follow-up to operated patients is commonly required, and interventions carried out in LMICs setting should be no exception. To comply with this, short missions should focus on common surgical procedures with short recovery time and low complication rates. Additionally, patients' education on the need to seek for medical care is still very scarce in LMICs, especially when it comes to treatable congenital anomalies or pediatric conditions. Participants to humanitarian missions should educate patients on risk factors and preventing measures for surgical diseases, notably when it comes to wound, skin or soft tissue infections (43).

Indeed, during our missions, we worked together with the host center to train parents on postoperative care and red flags requiring a timely access to healthcare facilities.

Lastly, one aspect that should be considered, mostly when organizing the delivery of material aids, is the different spectrum of pediatric surgical conditions found in LMICs when compared to the western ones. In these settings, there is little requirement for expensive tools to use in specialized surgeries; conversely, simple and effective solutions for emergency or general care are the main need. Infections requiring surgical treatment, traumas, and burns are by far the most frequent surgical conditions requiring hospitalization in LMICs, as pointed out by several authors (7, 44, 45). On one side this is due to an under-diagnosis of certain conditions caused by the lack of diagnostic tools, while on the other side to the living conditions of the population.

## Ethical Dilemmas Behind Humanitarian Missions

Over the past years, concerns are raising about the ethical issues inherited with humanitarian missions in LMICs. This reflects the growing awareness on patients' rights and the need for fair access to healthcare worldwide. Different ethical questions arise when profiling humanitarian aid: “How can we choose the patients who will benefit from the aid of a short-term mission?” and as a consequence, “Is It ethical to perform high-complexity interventions when more basic needs cannot be addressed?,” “How can our interventions be long-lasting?,” “Does our external help prevent the local healthcare system from growing?,” “Would our donated materials prevent the implementation of local factories and local know-hows?,” “Is informed consent really informed, considering the linguistic and cultural barriers between the patient and the western doctor?,” And again, “What will happen after help providers leave?”

As for all ethical dilemmas, it is not possible to find a univocal answer to these questions.

Throughout our relationship with Guinea Bissau, these challenges became more and more relevant for us. As a consequence, we started tailoring our missions with the aim of providing effective and sustainable aids.

As previously stated, many humanitarian missions that were started to perform complex interventions are now intended to build a solid foundation for pediatric surgery. Long-lasting improvements and accurate surgical plans are now key point during missions' planning.

As per the training of the local staff, the lack of specific educational programs is now quite evident and solutions to the paucity of workforce are being searched.

Referring to our experience, we did not only provide local staff training during our missions, but we also built up a program of continuative training via video conference systems. Dedicated lectures, tailored on local facilities, are given and mainly focus on perioperative care. When it comes to surgery, various surgical procedures are being detailed, with the final goal of creating the basis for in-place practical training and supervision.

Additional obstacles to an ethical provision of medical care involve linguistic and cultural barriers, which, most of the time, prevent an effective communication between western doctors and the local population. Subsequently, informed consent represent a real challenge for healthcare professional involved in humanitarian missions.

Visiting doctors often face several difficulties while illustrating the disease and the proposed therapeutic pathways to the patients; this may result in the provision of treatments without any certainty on the patients/parents understanding of the situation.

Besides linguistic barriers, western doctors are frequently unaware of the social backgrounds of the local population. As a result, predicting the consequences of major, life-changing interventions is a difficult task.

Patients and parents reaction before this situation can widely vary. Some people might blindly trust the visiting professionals, while others might prefer local illers and traditional treatments. A proper understanding between locals and western doctors represents a basepoint for the provision of informed consent and the creation of a trustful doctor-patient relationship. Similarly to HICs, ignoring the patients' social context is no longer acceptable.

In our experience, this was eased by the help of the local Chief of Surgery, who was trained at our Center and thus perfectly aware of the cultural background of both counterparts.

## Conclusions

In the coming years, a further expansion of the pediatric population in LMICs is expected; hence, the existing gap between the need for surgical and anesthetic treatment and the available therapeutic options will increase. Western countries will have to take action in order to decrease this gap. At present, HICs mostly act in LMICs through humanitarian missions, whose paradigm has started - and need - to change from a “short-term aid” to an intervention with long-lasting implications. In this perspective, the implementation of educational and training programs for local healthcare providers should be a top priority. Alongside, the population's awareness on the necessity to seek for medical care should be deployed, together with an eased access to health facilities. Humanitarian aids should always be tailored to the local situation and western countries should keep on working toward an ethical provision of human and medical resources.

## Author Contributions

RP and MG provided study conception and design, data collection, and writing – first draft and revised versions. DC provided writing – editing and revision. PG and CT provided study conception and design and writing – first draft and revised versions. All authors read and approved the final version of the article.

## Conflict of Interest

The authors declare that the research was conducted in the absence of any commercial or financial relationships that could be construed as a potential conflict of interest.

## Publisher's Note

All claims expressed in this article are solely those of the authors and do not necessarily represent those of their affiliated organizations, or those of the publisher, the editors and the reviewers. Any product that may be evaluated in this article, or claim that may be made by its manufacturer, is not guaranteed or endorsed by the publisher.

## Footnotes

^1^Available online at: http://healthforall.org/un-resolution/.

## References

[1] Meara JG Leather AJM Hagander L Alkire BC Alonso N Ameh EA. Global surgery 2030: evidence and solutions for achieving health, welfare, and economic development. Lancet. (2015) 386:569–624. 10.1016/S0140-6736(15)60160-X 25924834

[2] Ozgediz D Langer M Kisa P Poenaru D. Pediatric surgery as an essential component of global child health. Semin Pediatr Surg. (2016) 25:3–9. 10.1053/j.sempedsurg.2015.09.002 26831131

[3] Mock CN Donkor P Gawande A Jamison DT Kruk ME Debas HT. Essential surgery: key messages from disease control priorities, 3rd edition. Lancet. (2015) 385:2209–19. 10.1016/S0140-6736(15)60091-5 25662414 PMC7004823

[4] Butler EK Tran TM Nagarajan N Canner J Fuller AT Kushner A. Epidemiology of pediatric surgical needs in low-income countries. PLoS ONE. (2017) 12:e0170968. 10.1371/journal.pone.0170968 28257418 PMC5336197

[5] United Nations, Department of Economic and Social Affairs, Population Division. World Population Prospects: The 2017 Revision, Key Findings and Advance Tables. Working Paper No. ESA/P/WP/248. New York, NY: United Nations (2017). Available online at: https://population.un.org/wpp/publications/files/wpp2017_keyfindings.pdf

[6] UNICEF. Generation 2030 Africa 2.0. NEW York, NY: UNICEF (2017).

[7] Bickler SW Sanno-Duanda B. Epidemiology of paediatric surgical admissions to a government referral hospital in the Gambia. Bull World Health Organ. (2000) 78:1330–6. 11143193 PMC2560634

[8] Bickler SW Telfer ML Sanno-Duanda B. Need for paediatric surgery care in an urban area of The Gambia. Trop Doct. (2003) 33:91–4. 10.1177/004947550303300212 12680542

[9] Gupta S Shrestha S Ranjit A Nagarajan N Groen RS Kushner AL. Conditions, preventable deaths, procedures and validation of a countrywide survey of surgical care in Nepal. Br J Surg. (2015) 102:700–7. 10.1002/bjs.9807 25809125

[10] United Nations Inter-agency Group for Child Mortality Estimation (UNIGME). Levels & Trends in Child Mortality: Report 2019, Estimates Developed by the United Nations Inter-Agency Group for Child Mortality Estimation. New York, NY: United Nations Children's Fund (2019). Available online at: https://www.unicef.org/media/60561/file/UN-IGME-child-mortality-report-2019.pdf

[11] Bickler SW Kyambi J Rode H. Pediatric surgery in sub-Saharan Africa. Pediatr Surg Int. (2001) 17:442–7. 10.1007/s003830000516 11527185

[12] Mshelbwala PM Nwomeh BC. Paediatric surgery specialty and its relevance to Africa. Health. (2002) 80:829–35.

[13] Lozano R Naghavi M Foreman K Lim S Shibuya K Aboyans V. Global and regional mortality from 235 causes of death for 20 age groups in 1990 and 2010: a systematic analysis for the global burden of disease study 2010. Lancet. (2012) 380:2095–128. 10.1016/S0140-6736(12)61728-0 23245604 PMC10790329

[14] Shrime MG Bickler SW Alkire BC Mock C. Global burden of surgical disease: an estimation from the provider perspective. Lancet Glob Heal. (2015) 3(Suppl 2):S8–9. 10.1016/S2214-109X(14)70384-5 25926322

[15] Gosselin RA Thind A Bellardinelli A. Cost/DALY averted in a small hospital in Sierra Leone: what is the relative contribution of different services? World J Surg. (2006) 30:505–11. 10.1007/s00268-005-0609-5 16528459

[16] Holmer H Lantz A Kunjumen T Finlayson S Hoyler M Siyam A. Global distribution of surgeons, anaesthesiologists, and obstetricians. Lancet Glob Heal. (2015) 3(Suppl 2):S9–11. 10.1016/S2214-109X(14)70349-3 25926323

[17] O'Flynn E Andrew J Hutch A Kelly C Jani P Kakande I. The specialist surgeon workforce in east, central and Southern Africa: a situation analysis. World J Surg. (2016) 40:2620–7. 10.1007/s00268-016-3601-3 27283189

[18] The Lancet Commission on Global Surgery. Specialist Surgical Workforce (per 100,000 population). [Online] Available online at: https://data.worldbank.org/indicator/SHMEDSAOPP5

[19] Toobaie A Emil S Ozgediz D Krishnaswami S Poenaru D. Pediatric surgical capacity in Africa: current status and future needs. J Pediatr Surg. (2017) 52:843–8. 10.1016/j.jpedsurg.2017.01.033 28168989

[20] Chirdan LB Ameh EA Abantanga FA Sidler D Elhalaby EA. Challenges of training and delivery of pediatric surgical services in Africa. J Pediatr Surg. (2010) 45:610–8. 10.1016/j.jpedsurg.2009.11.007 20223329

[21] Lantz A Holmer H Finlayson SRG Ricketts TC Watters DA Gruen RL. Measuring the migration of surgical specialists. Surgery. (2020) 168:550–7. 10.1016/j.surg.2020.04.014 32620304

[22] Hagander LE Hughes CD Nash K Ganjawalla K Linden A Martins Y. Surgeon migration between developing countries and the United States: train, retain, and gain from brain drain. World J Surg. (2013) 37:14–23. 10.1007/s00268-012-1795-6 23052799

[23] Roy N. Global surgery: a view from the south. J Pediatr Surg. (2017) 52:203–6. 10.1016/j.jpedsurg.2016.11.006 27890315

[24] Ouro-Bang'na Maman A-F Kabore RAF Zoumenou E Gnassingbé K Chobli M. Anesthesia for children in Sub-Saharan Africa–a description of settings, common presenting conditions, techniques and outcomes. Paediatr Anaesth. (2009) 19:5–11. 10.1111/j.1460-9592.2008.02838.x 19076495

[25] Talabi AO Sowande OA Adenekan AT Adejuyigbe O Adumah CC Igwe AO. 10-year retrospective review of perioperative mortality in pediatric general surgery at Ile-Ife Hospital, Nigeria. J Pediatr Surg. (2018) 53:2072–6. 10.1016/j.jpedsurg.2018.03.005 29606409

[26] Organisation WH. Declaration of Alma Ata (1978). Available online at: https://www.who.int/teams/social-determinants-of-health/declaration-of-alma-ata#:~:text=The%20Alma%2DAta%20Declaration%20of,goal%20of%20Health%20for%20All.

[27] Backman G Hunt P Khosla R Jaramillo-Strouss C Fikre BM Rumble C. Health systems and the right to health: an assessment of 194 countries. Lancet. (2008) 372:2047–85. 10.1016/S0140-6736(08)61781-X 19097280

[28] Okoroh JS Chia V Oliver EA Dharmawardene M Riviello R. Strengthening health systems of developing countries: inclusion of surgery in universal health coverage. World J Surg. (2015) 39:1867–74. 10.1007/s00268-015-3031-7 25802236

[29] United Nations Institute for Training and Research. National Surgical, Obstetric and Anaesthesia Planning Manual. Geneva: United Nations Institute for Training and Research (2020). 10.5281/zenodo.3982869

[30] Pakistan's National Vision for Surgical Care. Available online at: https://www.globalsurgeryfoundation.org/pakistan-nsoap

[31] Nepal's NSOAP process. Available online at: https://www.globalsurgeryfoundation.org/nepal-nsoap

[32] Bickler SW Rode H. Surgical services for children in developing countries. Bull World Health Organ. (2002) 80:829–35. 10.1016/S0140-6736(15)60097-6 12471405 PMC2567648

[33] Budde-Schwartzman B Shwarzman O Lakhoo K Owusu F. Bringing specialist paediatric surgical care to the doorstep in rural Ghana: a mobile paediatric surgery clinic. Afr J Paediatr Surg. (2021) 18:195–200. 10.4103/ajps.AJPS_121_20 34341302 PMC8423172

[34] Jamison DT. Disease Control Priorities: improving health and reducing poverty. Lancet. (2018) 391:e11–4. 25662416 10.1016/S0140-6736(15)60097-6

[35] Global Initiative for Children's Surgery. Available online at: https://www.globalchildrenssurgery.org/

[36] Global Initiative for Children's Surgery. A model of global collaboration to advance the surgical care of children. World J Surg. (2019) 43:1416–25. 10.1007/s00268-018-04887-8 30623232 PMC7019676

[37] Farmer D Sitkin N Lofberg K Donkor P Ozgediz D. Surgical interventions for congenital anomalies. Dis Control priorities. (2015) 1:129–49. 26741013

[38] Small BM Hurley J Placidi C. How do we choose? J Clin Ethics. (2014) 25:308–10. 25525941

[39] Butler M Drum E Evans FM Fitzgerald T Fraser J Holterman AX. Guidelines and checklists for short-term missions in global pediatric surgery: recommendations from the American academy of pediatrics delivery of surgical care global health sub-committee, American pediatric surgical association global pediatric surgery. J Pediatr Surg. (2018) 53:828–36. 10.1016/j.jpedsurg.2017.11.037 29223665

[40] Ekenze SO Onumaegbu OO Nwankwo OE. The current status of international partnerships for child surgery in sub-Saharan Africa. Int Surg. (2014) 99:616–22. 10.9738/INTSURG-D-13-00244.1 25216431 PMC4253934

[41] Hubertus J Abera G Haileamlak A Siebeck M von Schweinitz D Wagner F. Establishment of a pediatric surgical unit at a university hospital in Eastern Africa. Child. (2021) 8:244. 10.3390/children8030244 33810100 PMC8005109

[42] Olivieri C Belay K Coletta R Retrosi G Molle P Calisti A. Preventing posterior sagittal anoplasty “cripples” in areas with limited medical resources: a few modifications to surgical approach in anorectal malformations. Afr J Paediatr Surg. (2012) 9:223–6. 10.4103/0189-6725.104724 23250244

[43] Kolkin J. A Physician's perspective on volunteering overseas… it is not all about sharing the latest technology. Front Surg. (2017) 4:77. 10.3389/fsurg.2017.00077 29457003 PMC5801311

[44] Gortan M Caravaggi P Brooks G Butoyi JMV Bambara S Nkurunziza J. Epidemiology of pediatric surgical conditions observed in a first-level hospital in Burundi. Front Pediatr. (2021) 9:681478. 10.3389/fped.2021.681478 34123976 PMC8192792

[45] Farmer DL. Audacious Goals - 2.0 The global initiative for children's surgery. J Pediatr Surg. (2017) 2:7. 10.1016/j.jpedsurg.2017.10.007 29173774

